# The effect of vitamin C on plasma volume in the early stage of sepsis in the rat

**DOI:** 10.1186/2197-425X-2-11

**Published:** 2014-03-06

**Authors:** Björn P Bark, Per-Olof Grände

**Affiliations:** Department of Anaesthesia and Intensive Care, Lund University and Lund University Hospital, SE-221 85 Lund, Sweden

**Keywords:** Plasma volume, Sepsis, SIRS, Vitamin C, Ascorbate, Ascorbic acid, Caecal ligation

## Abstract

**Background:**

Previous experimental studies have shown that vitamin C has several beneficial effects in sepsis and burns, such as decreased tissue oedema, improved endothelial barrier function and decreased transcapillary leakage of plasma markers. It has still not been investigated, though, if vitamin C has any impact specifically on plasma volume. The present study aims at testing the hypothesis that vitamin C decreases plasma volume loss in sepsis.

**Methods:**

Anaesthetized male adult Sprague-Dawley rats were used in this prospective randomized study. All experiments were carried out at a university hospital laboratory. Sepsis was induced by caecal ligation and incision. After 3 h, vitamin C was given either as a bolus dose (66 mg/kg) followed by a continuous infusion (33 mg/kg/h) (*n* = 9), or as a single bolus dose (200 mg/kg) (*n* = 9). A sham group (*n* = 9) underwent the same surgical procedure, but no vitamin C was given. Plasma volume was measured (^125^I-dilution technique) at baseline, at 3 h after end of initiation of sepsis and at the end of the experiment 3 h later. Arterial blood samples for analyses of electrolytes, blood gases, haematocrit and lactate were taken at the same time points.

**Results:**

There were no significant differences in plasma volumes or the physiological parameters analysed between any of the three groups at any time point. There was a significantly larger urine production in the single bolus dose group (200 mg/kg) compared to the sham group.

**Conclusions:**

Vitamin C treatment did not decrease the loss of plasma volume in the septic rat. The diuretic effect of vitamin C was in accordance with previous studies.

## Background

Vitamin C has been shown to have beneficial effects on the microcirculation in moderate sepsis in the rat [1, 2]. On a consensus meeting on vitamin C in acute endothelial pathophysiological conditions in 2006, it was concluded that there were arguments based on experimental studies for the hypothesis that high-dose vitamin C improves microvascular endothelial function in sepsis [3]. This hypothesis was further supported by some recent studies in septic mice. Thus, Fisher et al. showed that vitamin C has positive effects on various pathophysiological changes in sepsis, including the microvasculature of the lung [4, 5], and Zhou et al. showed that vitamin C decreases capillary leakage of different injected tracers [6].

Several experimental studies in different animal models have shown that vitamin C is also beneficial in burns by preventing capillary leakage, lymph flow and resuscitation fluid requirements [7–9]. In a more recent study, vitamin C treatment was shown to reduce the endothelial damage caused by transfusion of plasma from a burned donor rat [10]. Further, a human study showed a reduction in resuscitation volume with vitamin C treatment after severe burn [11]. In contrast, Aliabadi-Wahle et al. found no changes in microvascular permeability or in oedema formation when vitamin C was given after burn in the dog [12].

Even though not fully understood, suggested mechanisms behind the described beneficial effects of intravenous vitamin C treatment, in both sepsis and burns, are scavenging of reactive oxygen species, reduction of endothelial adhesion molecules and modulation of nitric oxide production [3, 13–15].

Sepsis, as well as burns, causes transcapillary leakage of plasma, reducing the circulating plasma volume [16, 17]. As discussed above, experimental studies have shown that vitamin C reduces local oedema and leakage of plasma markers. However, these findings do not necessarily reflect a decrease in plasma volume loss, and so far, no study has specifically investigated the effect of vitamin C treatment on plasma volume. In the present study, we therefore tested the hypothesis that vitamin C would reduce the loss of plasma volume in the early stage of sepsis. Treatment was initiated 3 h after induction of sepsis, a more clinically relevant time point than used in most studies found in the current literature, where treatment was started either before or closely after injury (e.g. sepsis, burns). Different dose regimes have been used in previous studies. Beneficial effects on microcirculation have been shown with low-dose treatment [1, 2], but it seems that higher doses are needed to counteract microvascular leakage. We chose to compare two different treatment regimes, both previously shown to be effective to prevent capillary leakage - one with a small bolus dose followed by a continuous infusion [9, 10] and one with a high bolus dose as single treatment [4, 6]. A sham group that underwent the same surgical procedure, but received no treatment, was also included in the study.

## Methods

### Anaesthesia and set-up

The study was approved by the Ethical Committee for Animal Research at Lund University, Sweden (application no. M180-10). The animals were treated in accordance with the guidelines of the National Institutes of Health for Care and Use of Laboratory Animals. Male adult Sprague-Dawley rats were used, weighing 337 ± 26 g (mean ± SD). Anaesthesia was induced using a covered glass container with a continuous supply of 5% isoflurane in air (Forene® 100%; Abbot Scandinavia AB, Solna, Sweden), in which the animals were placed. After induction, the animals were removed from the container, and anaesthesia was maintained with 1.5% to 1.8% isoflurane in air using a mask, while tracheostomy was performed. Thereafter, the animals were connected to a ventilator (Ugo Basile; Biological Research Apparatus, Comerio, Italy) and ventilated in a volume-controlled mode with a positive end expiratory pressure of 4 cm H_2_O. End-tidal PCO_2_ was continuously monitored (Capstar-100; CWE, Ardmore, PA, USA). Anaesthesia was maintained with 1.5% to 1.8% isoflurane in air throughout the experiment. Body temperature, measured rectally, was kept at 37.1°C to 37.3°C using a feedback-controlled heating pad. The left femoral artery was cannulated to monitor arterial blood pressure and to obtain blood samples for analysis of electrolytes, haematocrit, lactate, arterial blood gases (I-STAT; Abbot Point of Care Inc, Abbot Park, IL, USA) and plasma volumes. The left femoral vein was cannulated and used for infusions, and kept open with a continuous infusion of saline at 0.2 μL/min. The right internal jugular vein was cannulated and used for injection of ^125^I-albumin for plasma volume measurements. At the end of the experiments, the animals were sacrificed with an intravenous injection of potassium chloride.

### Experimental procedure

A well-established rat model of severe sepsis was used [16, 18]. A longitudinal midline skin incision in the abdominal wall with diathermia was performed, followed by laparotomy by incision along the linea alba. After ligation just below the ileocaecal valve, an incision of 1 cm in length was made in the caecum, allowing leakage of faeces into the abdominal cavity, thereby inducing sepsis/systemic inflammatory response syndrome (SIRS). The abdominal wall and the skin were then closed with clips. There was no bleeding during the experiment.

### Plasma volume

Plasma volume (PV) was determined with a reliable and established technique, shown to produce reproducible and reliable results [16, 19–21]. As described previously [16], PV was determined by measuring the radioactivity in 100 μL of plasma taken 5 min after an intravenous injection of human ^125^I-albumin (0.5 mL) with a known amount of activity. The increase in radioactivity was calculated by subtracting the activity in a blood sample taken just before the injection from that taken 5 min after the injection, thereby adjusting for any remaining radioactivity from previous measurements. To calculate the amount of radioactivity given, the remaining activity in the emptied vial, syringe and needle used was measured and subtracted from the total activity in the prepared dose. Sources of error are small with the technique used. Free iodine was measured regularly following precipitation with 10% trichloroacetic acid and was found to be less than 2.0% in the prepared samples. Radioactivity was measured with a gamma counter (Wizard 1480; LKB-Wallac, Turku, Finland).

### Experimental protocol

In this study, we evaluated the effect of intravenous vitamin C on plasma volume in the early stage of sepsis in the rat. The septic rats were divided into three groups: a bolus + infusion group (the B + I group, *n* = 9), a bolus group (the B group, *n* = 9) and a sham group (the S group, *n* = 9). Animals that did not show a decrease in PV 3 h after the preparation were considered to be non-septic and were excluded from the study. These animals and animals that died before the end of the experiment were replaced with new animals.

**Figure 1:**
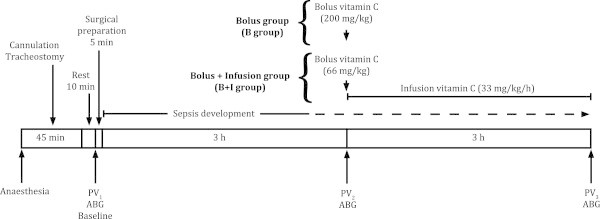
**Time scale of the experiment.** PV_1_, plasma volume at baseline; PV_2_, plasma volume 3 h after surgical preparation just before the start of treatment; PV_3_, plasma volume at the end of the experiment; ABG, arterial blood sample for analysis of blood gases, hematocrit, lactate and electrolytes.

After cannulation and surgical preparation, the animals were left undisturbed for 3 h, a time period previously shown to be sufficient for systemic inflammation and plasma leakage to develop [16]. Three hours after surgical preparation, the treatment was initiated. The B + I group received an intravenous injection of ascorbic acid (2,3-didehydro-l-threo-hexono-1,4-lactone, Askorbinsyra 100 mg/ml, APL, Stockholm, Sweden) of 66 mg/kg, followed by an infusion of 33 mg/kg/h during the rest of the experiment (Figure 1). In previous studies, this dose regime has been shown to be effective in decreasing microvascular permeability after burns in the rat [9, 10]. The B group received a single intravenous bolus injection of 200 mg/kg of ascorbic acid (Figure 1), previously shown to be effective in septic mice [4, 6]. The S group received no treatment, as it was meant to represent a non-treatment situation.

Plasma volumes were measured at baseline, at 3 h after the end of surgical preparation and at the end of the experiment another 3 h later. Blood samples for measurement of arterial pH, PCO_2_, PO_2_, lactate, haematocrit, sodium and potassium were taken at the same time points.

Urine was collected in a glass vial placed at the external meatus of the urethra throughout the whole experiment, and the bladder was emptied by external compression at the end of the experiment.

### Statistical analysis

Statistical analyses were performed with GraphPad Prism software version 5.0c for Mac OS X (GraphPad Software, San Diego, CA, USA). Physiological data and plasma volumes were compared using two-way ANOVA for repeated measures followed by Bonferroni post hoc test and unpaired two-tailed Student's *t* test. Urine productions were compared using unpaired two-tailed Student's *t* test. Differences were considered significant when *p* < 0.05. To achieve a statistical power of 90% with a difference in PV (PV_3_ − PV_2_) between groups of 4 mL/kg, the calculated sample size for each group was 9. All data were normally distributed. The results are presented as mean ± SD.

## Results

Five animals died before the end of the experiment, evenly distributed between the groups. Eleven animals were considered to be non-septic and were excluded from the study, as they did not show a decrease in PV 3 h after the preparation.

### Physiological data

Data for sodium (Na^+^), potassium (K^+^), haematocrit (Hct), lactate (Lac), pH, PaCO_2_ and PaO_2_, from arterial blood samples taken at baseline, at 3 h after the end of surgical preparation and at the end of the experiment 3 h later are summarized in Table 1. Data for arterial blood pressure are presented in Table 2. There were no significant differences between the groups in blood pressure or any of the parameters analysed at any time point. There was a significant increase in potassium and lactate levels, and a decrease in pH and PaCO_2_ in all groups at the end of the experiments compared to baseline (Table 1).

**Table 1:** **Physiological data**

	Na^+^(mmol/L)	K^+^(mmol/L)	Hct (%)	Lac (mmol/L)	pH	PaCo_2_(kPa)	PaO_2_(kPa)
Bolus + infusion group (*n* = 9)							
Baseline	136 ± 2	4.9 ± 0.3	42 ± 1	2.4 ± 0.3	7.48 ± 0.04	4.9 ± 0.4	11.0 ± 0.8
3 h after prep	133 ± 3	5.3 ± 0.6	45 ± 3	2.7 ± 0.4	7.44 ± 0.03	4.9 ± 0.5	10.8 ± 0.7
End of experiment	135 ± 2	6.2 ± 0.8***	50 ± 5**	3.2 ± 0.7**	7.43 ± 0.04*	4.2 ± 0.4**	11.4 ± 0.5
Bolus group (*n* = 9)							
Baseline	137 ± 2	4.4 ± 0.3	42 ± 3	2.3 ± 0.5	7.48 ± 0.03	5.0 ± 0.4	11.1 ± 0.6
3 h after prep	133 ± 2	5.2 ± 0.3	45 ± 3	2.9 ± 0.5	7.44 ± 0.02	4.9 ± 0.4	11.4 ± 0.5
End of experiment	135 ± 2	5.8 ± 0.9***	50 ± 4***	3.4 ± 0.8**	7.43 ± 0.04**	3.7 ± 0.7***	12.5 ± 1.3
Sham group (*n* = 9)							
Baseline	136 ± 2	4.8 ± 0.6	43 ± 2	2.3 ± 0.2	7.48 ± 0.05	4.9 ± 0.5	11.0 ± 0.9
3 h after prep	134 ± 2	5.1 ± 0.6	45 ± 1	2.6 ± 0.3	7.45 ± 0.03	4.8 ± 0.3	11.0 ± 1.0
End of experiment	134 ± 2	5.9 ± 0.6**	49 ± 2***	2.9 ± 0.7*	7.44 ± 0.03*	4.1 ± 0.3***	12.0 ± 0.7

Data (mean ± SD) for sodium (Na^+^), potassium (K^+^), haematocrit (Hct), lactate (Lac), pH, arterial partial pressure of carbon dioxide (PaCO_2_) and arterial partial pressure of oxygen (PaO_2_). There were no significant differences between any of the parameters analysed, between groups at any time points. Two-way ANOVA for repeated measures followed by Bonferroni post hoc test was used for the statistical analyses. There were significant differences in K^+^, Hct, Lac, pH and Paco_2_ between baseline and the end of the experiments in all groups. Unpaired two-tailed Student's *t* test was used for the analyses. *p < 0.05, **p < 0.01, ***p < 0.001.

**Table 2:** **Blood pressure**

	Baseline	3 h after surg prep	1.5 h after start of treatment	3 h after start of treatment
Bolus + infusion group (*n* = 9)	96 ± 11	92 ± 12	99 ± 14	96 ± 13
Bolus group (*n* = 9)	88 ± 15	91 ± 12	92 ± 16	90 ± 13
Sham group (*n* = 9)	95 ± 12	90 ± 11	94 ± 7	93 ± 7

Data (mean ± SD) for mean arterial blood pressure (mmHg) at baseline, at 3 h after the surgical preparation and at 1.5 and 3 h after the start of treatment. There were no differences between any of the groups at any time points. Two-way ANOVA for repeated measures followed by Bonferroni post hoc test was used for the statistical analyses.

### Plasma volume

**Figure 2:**
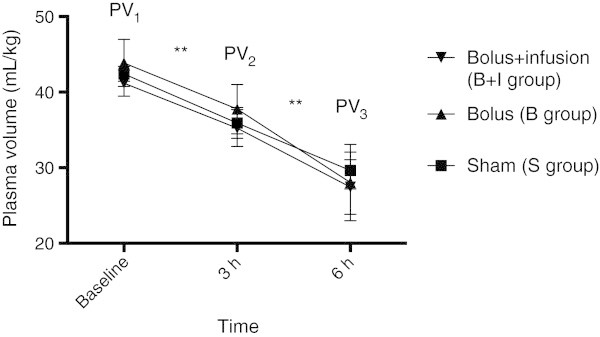
**Plasma volumes.** Plasma volumes at baseline (PV_1_), at 3 h after the surgical preparation just before the start of treatment (PV_2_) and at the end of the experiment (PV_3_). There was no significant difference between any of the groups at any time points. There was a significant difference between PV_1_ and PV_2_, and PV_2_ and PV_3_ for all groups. Two-way ANOVA for repeated measures followed by Bonferroni post hoc test was used for the statistical analyses (**p < 0.01).

Plasma volumes at baseline, at 3 h after the end of the surgical preparation and at the end of the experiment 3 h later were 41.2 ± 1.7 mL/kg, 35.2 ± 2.4 mL/kg and 27.4 ± 3.6 in the B + I group; 43.9 ± 3.1 mL/kg, 37.8 ± 3.3 mL/kg and 28.0 ± 5.0 mL/kg in the B group; and 42.4 ± 1.0 mL/kg, 35.9 ± 2.0 mL/kg, and 29.6 ± 2.4 mL/kg in the S group (Figure 2). There were no significant differences in PV between the three groups at any time point. There was a significant reduction in plasma volume in all three groups 3 h after the end of the surgical preparation (PV_2_) compared to baseline (PV_1_), and at the end of the experiment (PV_3_) compared to 3 h after the end of the surgical preparation (PV_2_) (*p* < 0.01) (Figure 2).

### Urine production

**Figure 3:**
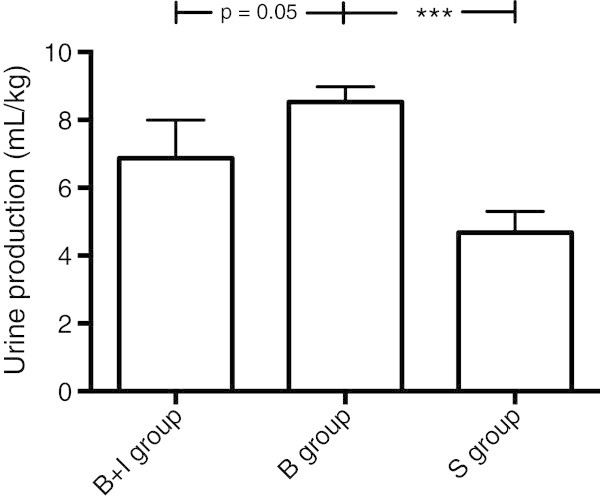
**Urine production.** Data for urine production (mL/kg) from the end of surgical preparation to the end of the experiment. There was a significantly larger urine production in the B group compared to the S group. Student's *t* test was used for the statistical analyses (***p < 0.001).

Urine production from the end of surgical preparation to the end of the experiment was 6.9 ± 3.4 mL/kg in the B + I group, 8.5 ± 1.4 mL/kg in the B group and 4.7 ± 1.8 mL/kg in the S group. The urine production was significantly larger in the B group than in the S group (*p* < 0.001) (Figure 3).

## Discussion

The two investigated treatment regimes of vitamin C had no effect on plasma volume loss or any of the physiological parameters analysed in the early stage of sepsis in the present study in the rat. The larger urine production in the B group was in accordance with previous studies, both in humans and in dogs, showing a diuretic effect of vitamin C [22, 23]. It has been suggested [23] that this effect is due to an increase in glomerular filtration, although the exact mechanism of action is unclear. However, as urine represents loss of fluid from the entire extracellular space, meaning that only 20% to 25% of the volume is lost from the PV, the larger urine production in the B group will only have had a minor effect on the PV (0.8 to 1.0 mL/kg). A previous study on anaesthetised rats with artificial ventilation has shown that perspiration during this time period has no effect on plasma volume [24]. This means that the plasma volume loss in the present study must represent tissue oedema.

The higher potassium concentrations and lower PaCO_2_ and pH at the end of the experiments in all groups are compatible with sepsis/SIRS-induced cell destruction, increased lactate production and subsequent compensatory hyperventilation (Table 1).

The ^125^I-dilution technique for measurement of plasma volume is well established and reliable with small potential errors as described in detail previously [16, 19–21]. There might have been overestimation of plasma volume because of transcapillary escape of radioactive albumin during the 5-min period between injection of the tracer and collection of the blood sample. However, errors will have no or minor influence on the conclusions made, as they would have been of the same magnitude in all groups.

By its vasodilatory effect, isoflurane might have increased the transcapillary plasma leakage by an increase in capillary pressure. This increase, however, must have been of the same magnitude in all groups and will therefore have no influence on the conclusions made.

While previous studies have shown beneficial effects of vitamin C, such as reduced transcapillary leakage, reduced lymph flow and reduced oedema formation, using the same treatment regimes that were used in the present study [4, 6, 9, 10], we could not demonstrate any effects on the plasma volume loss.

As mentioned in the ‘Background’, in most of the experimental studies listed above showing beneficial effects of vitamin C, treatment was initiated either prior to injury or shortly thereafter. The fact that we started the treatment 3 h after injury might explain our negative results. Our negative results may also be partly explained by the fact that caecal ligation and incision used in the present study probably resulted in a more severe sepsis than in the caecal ligation and puncture model used in many other studies. We also chose to evaluate the effect on plasma volumes 3 h after initiation of treatment, a time period shorter than in most other previous studies, and it cannot be excluded that this might have contributed to our negative results. In one study [6], treatment with vitamin C (200 mg/kg) was initiated 3 h after injury, as in the present study, and they demonstrated a positive effect, in terms of reduced capillary leakage of Evans blue after 12 h in the septic mouse. This study was performed in rats, and our results might therefore not be directly transferred to man. For example, in contrast to man, rats are able to synthesize vitamin C.

## Conclusions

In conclusion, the present study did not confirm our hypothesis, as intravenous vitamin C treatment initiated 3 h after induction of sepsis had no effect on the loss of plasma volume, or any of the physiological parameters analysed, in the early stage of sepsis in the rat. High-dose vitamin C caused an increase in urine production.
